# Phenotypic Markers Reflecting the Status of Overstressed Tea Plants Subjected to Repeated Shade Cultivation

**DOI:** 10.3389/fpls.2020.556476

**Published:** 2020-11-06

**Authors:** Hiroto Yamashita, Yasuno Tanaka, Keisuke Umetsu, Sakurako Morita, Yoshiki Ono, Toshikazu Suzuki, Tetsuyuki Takemoto, Akio Morita, Takashi Ikka

**Affiliations:** ^1^Faculty of Agriculture, Shizuoka University, Shizuoka, Japan; ^2^United Graduate School of Agricultural Science, Gifu University, Gifu, Japan; ^3^Tea Research Center, Shizuoka Prefectural Research Institute of Agriculture and Forestry, Kikugawa, Japan; ^4^Agriculture and Forestry Technology Department, Kyoto Prefectural Agriculture, Forestry and Fisheries Technology Center, Uji, Japan; ^5^Institute for Tea Science, Shizuoka University, Shizuoka, Japan

**Keywords:** tea plants, shading, photosynthesis, non-structural carbohydrate, metabolome

## Abstract

Shade cultivation is a traditional Japanese tea cultivation method in which the shoot buds are shaded for several weeks. This technique is increasingly used for green tea production because it produces tea of high quality (as indicated by umami and nutritional content) and commands high prices. However, given that shaded tea plants are grown under low-light stress, concerns exist regarding damage to tea plants caused by repeated shade cultivation. To understand basic physiological responses and accumulative changes in photosynthetic ability and metabolites of tea plants subjected to repeated shading, we performed a pot experiment on immature tea plants grown in a growth chamber subjected to repeated shading treatments. The results demonstrated that shade cultivation caused a decrease in non-structural carbohydrate content and an increase of several degrees in leaf surface temperature, reflecting transpiration through the leaf stomata, as a result of a reduction in photosynthetic ability. An increase of several degrees in canopy temperature and a reduction in photosynthetic ability in the field in the mid-summer season was also observed in overstressed tea plants subjected to repeated shading. Metabolomic analysis identified several candidate biomarkers, such as citrulline and glycine betaine, that were significantly changed in individuals affected by shade cultivation. These physiological changes may be an indicator of the stress status of tea plants grown under repeated shade cultivation.

## Introduction

Japanese green tea, one of the most popular non-alcoholic beverages worldwide, is produced from unfermented (steamed) tea leaves in Japan. Sencha, a type of Japanese tea, contains many functional components, such as amino acids and catechins, and is widely consumed because of its health benefits (Namita et al., 2012). In recent years, *Matcha* and *Gyokuro*, which are tea types with enhanced umami and green color (Goto et al., 1996; Horie et al., 2018), have become globally popular, and demand for these products is increasing. The production and export of Japanese green teas have gradually increased (Ministry of Agriculture Forestry And Fisheries of Japan, 2016). However, the current supply is insufficient to meet the increase in demand; therefore, improvement of the production system for high-quality Japanese green teas, especially *Matcha* and *Gyokuro*, is necessary.

The production of *Matcha* and *Gyokuro* is achieved using a traditional Japanese system termed “shade cultivation.” Shade cultivation is a method that reduces the incident irradiation on tea plants by 60–98% (typically 85%) by covering the tea field or the tea plants directly with shading material, such as synthetic black cloth. In general, shading is applied when two leaves have developed from the majority of new buds in the season of the first crop (or flush, in spring) and/or the season of the second crop (summer) for about 10–30 days. The morphological and chemical phenotypes of new shoots of shaded tea plants change markedly under shade cultivation (Sano et al., 2018). The most typical response to shading treatment is the darker green color of leaves owing to increased chlorophyll content (Sano et al., 2018). Previous studies (Chutani and Takewaka, 2006; Kobayashi et al., 2011; Matsunaga et al., 2016; Sano et al., 2018) have additionally revealed that shading treatment causes an increase in the contents of amino acids, especially theanine, and a decrease in the contents of catechins, especially epigallocatechin. The contents of free amino acids and chlorophyll are extremely important determinants of tea quality and price. The price of Japanese green tea tends to be proportional to the amino acid content (Mukai et al., 1992). Hence, tea production by shade cultivation, which improves these qualities, is actively promoted in Japan.

Light is the most important factor influencing plant growth and productivity. Normal plant growth requires the optimal light intensity because excessively high or low light results in photo-inhibition and light deficiency, respectively, with plant growth consequently severely restricted. Under low light intensity, reductions are observed in net photosynthetic rate (Bjorkman, 1972) as well as the contents of non-structural carbohydrates (NSCs), such as starch and sugars, the primary photosynthetic assimilation products (Robbins and Pharr, 1987). The productivity of tea plants also depends on the amount of NSCs in sink organs, such as shoots and/or roots (Suzuki et al., 2013b). Given that tea plants are a perennial crop, accumulative damage caused by prolonged exposure to low-intensity light from repeated shading is a risk. Takemoto and Hayashi (2019) have reported that low-light stress caused by repeated shade cultivation leads to a decrease in the growth of new shoots in the first crop season and an increase in the canopy surface temperature in mid-summer.

Plants show a tremendous capacity to adjust not only their morphology and physiology, but also their metabolism, in response to changes in light intensity. To understand this metabolic regulation and responses to environmental factors, metabolomics is a powerful tool that is useful for diagnostic analyses of plants (Schauer and Fernie, 2006). In plant metabolomics research, several techniques, such as gas chromatography–mass spectrometry, liquid chromatography–mass spectrometry, capillary electrophoresis–mass spectrometry, and nuclear magnetic resonance spectroscopy, have been developed and commonly applied (Obata and Fernie, 2012). In particular, light intensity-dependent metabolic shifts have been observed in the leaves of Arabidopsis (Jänkänpää et al., 2012). In addition, metabolomic analysis has revealed that genetic manipulation of the content of Rubisco, the crucial enzyme that catalyzes CO_2_ fixation during photosynthesis, broadly affects carbon (C) and nitrogen (N) metabolism in rice (Suzuki et al., 2012). Metabolomic analytical approaches have great potential for acquiring comprehensive information on metabolic networks and for identifying biomarkers associated with abiotic stresses (Obata and Fernie, 2012). Biomarkers enable prediction of phenotypic traits before such features become apparent and are therefore valuable tools for both fundamental and agricultural science.

In this study, to clarify the accumulative effects of repeated shading on growing tea plants, we first conducted a pot experiment using immature tea plants repeatedly subjected to low-intensity light in a growth chamber. We measured their photosynthetic ability at approximately 5-day intervals as well as chlorophyll and NSC contents after each shading treatment. We then applied repeated shading treatments to mature tea plants cultivated in the field and measured the same NSC content and photosynthetic indicators as in the pot experiment. Finally, a metabolomic analysis of the trunks of the field-treated plants was conducted to identify candidate biomarkers of the repeated-shading-induced stress status of tea plants.

## Materials and Methods

### Plant Materials and Growth Conditions

To evaluate the physiological response of tea plants to repeated shading, a pot experiment was first conducted as a basic model experiment. Two-year-old rooted cuttings of tea (*Camellia sinensis* L.) “Yabukita,” a leading Japanese green tea cultivar, were transplanted to 1/5000 a Wagner pots containing tea field soil collected in Makinohara City, Shizuoka, Japan. Before transplanting, 0.18 g N, 0.04 g P_2_O_5_, and 0.08 g K_2_O were mixed into the soil in each pot as basal fertilizer. Prior to repeated shading treatments, the plants were cultivated in a greenhouse in accordance with conventional practices at Shizuoka University, Shizuoka, Japan. Each pot was irrigated daily with 200 mL tap water.

After the majority of new shoots had developed two leaves, each plant was transferred to growth chambers (LPH-411SPC, Nippon Medical and Chemical Instruments, Osaka, Japan) for application of shading treatments. The light intensity in the growth chambers was set to a photosynthetic photon flux density (PPFD) of 500 μmol m^–2^ s^–1^ (control) or 75 μmol m^–2^ s^–1^ (85% shading). The other environmental variables were uniform in both chambers: temperature of 25°C, relative humidity of 65 ± 5%, CO_2_ concentration of 400 ppm, and a photoperiod of 14/10 h (day/night). Each pot was irrigated daily with 200 mL tap water. The amount of irrigation was set at conditions similar to a main tea cultivation region, Shizuoka, Japan, to avoid drought stress. After 14 days, the third or fourth leaves that had developed on the new shoots were harvested, with the lower leaves left in place. Subsequently, all plants were cultivated under the control light intensity (PPFD of 500 μmol m^–2^ s^–1^) until the next shading treatment. After the development of two new leaves (NL) on the majority of new shoots, the shade-treated plants were subjected to the same shading treatment applied previously. Each experiment was carried out with three biological replicates. After the first and second shading treatments, one plant of each treatment replicate was sampled. Finally, except for one stunted individual, a third shading treatment was carried out with three biological replicates. The shading treatments were applied three times as shown in Supplementary Figure 1A.

In this experiment, the lowest leaves remaining on the first new shoots after harvesting following the first shading treatment were defined as mature leaves (ML). Subsequently, these ML were used for the measurement of photosynthetic ability and leaf temperature at approximately 5-day intervals. After the second shading treatment, one plant per replicate was sampled and washed with tap water to remove soil particles from the roots. These plants were divided into leaves, stems, and roots, and each part was separated further into young and mature organs as follows (Supplementary Figure 1B): NL, new stems (NS), ML, mature stems (MS), very mature leaves (VML), branches (BR, φ < 10 mm), trunks (TR, φ > 10 mm), lignified roots (LR), and new roots (NR). Each organ was immediately frozen in liquid nitrogen and stored at −80°C until metabolomic analysis. After the third shading treatment, the remaining plants from the three replicates of each treatment were harvested as described above. Each fresh sample was weighed to record the fresh weight and then reweighed after freeze-drying to record the dry weight. Dry samples were ground to a fine powder and stored at room temperature in a desiccator for subsequent chemical component analysis.

To evaluate the effect of repeated shading on starch content as a C resource in mature tea plants, a field experiment was conducted in the tea field of the Tea Research Center, Shizuoka Prefectural Research Institute of Agriculture and Forestry, Kikugawa City, Shizuoka, Japan. In this field experiment, the area of tea “Yabukita” ridges was 5 m long × 1.8 m wide per treatment. The conventional tea cultivation practice (no shading) was used as the control, and the following three treatments were applied. For shading pattern 1 (SP1), representing the Japanese *Kabusecha* (from “*Kabuse*,” Japanese for “cover”) production style, ridges were shaded with 85% synthetic black cloth (85P, Dio Chemicals, Tokyo, Japan) after the development of two NL for 15 days in the first crop season and for 10 days in the second crop season, respectively. For shading pattern 2 (SP2), representing the Japanese *Tencha* (*Matcha*) production style, ridges were shaded with 85% synthetic black cloth (85P, Dio Chemicals) after the development of two NL for 30 days in the first crop season and for 15 days in the second crop season, respectively. For shading pattern 3 (SP3), representing the Japanese White leaf tea production style described by Kobayashi et al. (2011), ridges were shaded with three layers of 98% synthetic black cloth (2200, Dio Chemicals), prepared to approximate 100% synthetic black cloth, after the development of two NL for 15 days in the first crop season and for 10 days in the second crop season, respectively. Each shading experiment comprised three replicates in the first and second crop seasons. Shading materials directly covered the tea canopies as described by Sano et al. (2018). The shading treatments were conducted in 2016 and 2017. For measurement of starch content, the trunks of tea plants (φ > 10 mm) were harvested as follows: in 2016, after the second crop season and the autumn season; in 2017, at bud opening in the first crop season, after the first crop season, and after the second crop season. The branches of tea plants (φ < 10 mm) were harvested as follows: in 2017, at bud opening in the first crop season, after the first crop season, and after the second crop season.

To evaluate the effect of long-term repeated shading, we used a Japanese *Tencha* (*Matcha*) production field exhibiting accumulated damage from repeated shading as previously reported in Takemoto and Hayashi (2019). The detailed experimental design is shown in Supplementary Figure 3. The ridges were shaded with 85% synthetic black cloth (85P, Dio Chemicals) for 30 days in the first crop season and for 20 days in the second crop season for 6 consecutive years (“6y-shading”), namely, from 2011 to 2016. Shading materials directly covered the tea canopies as described above. These tea ridges had already been reported to show reduced yield and a higher canopy temperature compared with those of the control ridges (no shading) in mid-summer (Takemoto and Hayashi, 2019). In 2017, these tea ridges were returned to conventional tea cultivation practice (no shading) to evaluate the accumulated damage caused by prolonged shading. We also used additional tea ridges that were shaded with 85% black cloth (85P, Dio Chemicals) for 30 days only in the first crop season for 3 consecutive years (“3y-shading”), from 2014 to 2016. Neighboring tea ridges subjected to conventional tea cultivation practice (no shading) were used as controls (“3y-control” and “6y-control”). These experiments were conducted with three replicates. Yield components were assessed in the first crop season. At the bud-opening stage during the first crop season and the new bud-development stage in mid-summer, we additionally harvested ML, BR (φ < 10 mm), and TR (φ > 10 mm) for measurement of starch content, and the trunks were subjected to metabolomic analysis. Because we were unable to harvest the trunk of the “6y-control” at the bud-opening stage, we used the “3y-control” trunk at the same stage as a substitute. On sunny days in mid-summer, we measured the photosynthetic ability of five randomly selected ML and the canopy temperature of each treated tea ridge.

### Yield and Yield Components in the Mature Tea Field

Yield and yield components were evaluated by conducting a quadrat survey following the method of Sano et al. (2018). Theoretical yield (kg 10 a^–1^) in the tea field was calculated from the fresh weight of young shoots in each quadrat (200 mm × 200 mm).

### Measurement of Chlorophyll Content

Chlorophyll a and b were extracted from finely ground powder (5 mg) of freeze-dried leaf samples with 5 mL *N*,*N*′-dimethylformamide. After incubation for 24 h at 4°C under dark conditions to allow complete decolorization, the samples were centrifuged at 2000 × *g* for 30 min, and the absorbance of the supernatant was measured at 663.8 and 646.8 nm with a spectrophotometer (UV-1800, Shimadzu, Kyoto, Japan). Chlorophyll *a* and *b* contents were calculated using the equation of Porra et al. (1989).

### Measurement of Starch and Sugar Contents

Extraction and quantification of starch in the tea tissues were conducted in accordance with the methods of Suzuki et al. (2013a). Fine powder (100 mg) from the freeze-dried samples was added to 5 mL ultra-pure water, and starch in the sample was gelatinized in boiling water for 30 min. After cooling to room temperature, the samples were centrifuged at 2000 × *g* for 15 min, and the supernatant was collected. An additional 5 mL ultra-pure water was added to the residue, and the supernatant was collected after the extraction steps were repeated. Next, 3.8 mL of 6 N HCl solution and 0.2 mL of 0.025 M iodine solution were added to 1 mL of the properly diluted supernatant to prevent saturation. The solution was mixed on a vortex mixer, and the absorbance was measured at 600 nm using a spectrophotometer (UV-1800, Shimadzu). Pure starch from potato (Merck, Darmstadt, Germany) was used as a standard.

Extraction and quantification of sugar in the tea tissues was conducted in accordance with the methods of Anan et al. (1981) with some modifications. Fine powder (100 mg) of the freeze-dried samples was added to 5 mL of 80% EtOH. The suspension was incubated in a water bath at 80°C for 15 min, then shaken for 30 min on a shaker (Recipro Shaker R-I, Taitec, Saitama, Japan) at room temperature. The samples were centrifuged at 2000 × *g* for 15 min, and the supernatant was collected. Next, 2.5 mL of 80% EtOH was added to the residue, and the supernatant was collected after the repetition of the extraction steps. The EtOH in the supernatant was evaporated using a dry thermal bath system (MG-2300, EYELA, Tokyo, Japan). One milliliter of 5% ZnSO_4_ and 1 mL 0.3 N Ba(OH)_2_ were added to the evaporated supernatant, and the solution volume was adjusted to 20 mL using ultra-pure water. After filtration through filter paper (No. 6, Advantec, Tokyo, Japan), glucose, fructose, and sucrose concentrations, representing the sugar concentration in the filtrate, were measured by high-performance liquid chromatography (HPLC). The HPLC system (Shimadzu, Tokyo, Japan) consisted of the following components: LC-9A pump, CTO-6A column oven, RID-6A refractive index detector, CBM-20A system controller, and SIL-20AC auto-sampler. The HPLC conditions were as follows: injection volume, 20 μL; column, 4.6 × 250 mm Sugar-D (Nacalai Tesque, Kyoto, Japan); column oven temperature, 35°C; mobile phase, 75% acetonitrile; and flow rate, 1 mL min^–1^. The mobile phase was eluted for 15 min per sample.

### Mineral Analysis

Fine powder (20 mg) of the freeze-dried samples was digested in 2 mL of 60% HNO_3_ at 110°C in a DigiTUBE tube (SCP Science, Montreal, Canada) for approximately 2 h. After the samples had cooled to room temperature, 2 mL of 60% HClO was added, and the samples were heated at 110°C for approximately 2 h. Once digestion was complete, the samples were cooled and the solution volume was adjusted to 10 mL by the addition of ultra-pure water. The total concentrations of magnesium (Mg) and iron (Fe) were measured at 285.213 and 259.940 nm, respectively, using an inductively coupled plasma–optical emission spectrometer (iCAP 7400, Thermo Fisher Scientific, Waltham, MA, United States).

Total N and C contents were measured by dry combustion using an NC analyzer (SUMIGRAPH NC-95A, Sumika Chemical Analysis Service, Tokyo, Japan). Acetanilide was used as a standard for total C and N analyses.

### Evaluation of Photosynthetic Ability

Photosynthetic rate, stomatal conductance, and transpiration rate were measured with a portable photosynthesis system (Li-6400XT, Li-Cor, Lincoln, NE, United States). The conditions in the leaf chamber were set as follows: PPFD, 1500 μmol m^–2^ s^–1^; CO_2_ concentration, 400 μmol mol^–1^; and temperature, 25°C. Photosynthesis was measured from 10:00 to 14:00. The net photosynthetic rate was expressed as the rate of CO_2_ uptake.

### Measurement of Leaf Surface and Canopy Temperatures

Leaf surface temperature was measured using an infrared thermograph (TH6300R, Nippon Avionics, Tokyo, Japan) as described previously (Takemoto and Hayashi, 2019). Thermal images of the leaf surface of immature tea plants (pot cultivation) were photographed with the thermograph using an individual leaf around midday in a growth chamber. Thermal images of the canopy of mature tea plants (tea field) were photographed with the thermograph from three perspectives—at eye level, looking downward at a 30° angle, and targeting the canopy from a 1.5-m distance—around midday on a sunny day. The air temperature for each tea ridge in the field in the 3y-control, 3y-shaded, 6y-control, and 6y-shaded treatments was 36.1 ± 0.4, 36.7 ± 0.2, 35.2 ± 0.6, and 34.4 ± 0.5°C, respectively. Canopy temperature was calculated using thermal image analysis software (InfReC Analyzer NS9500 Standard, Nippon Avionics).

### Metabolomic Analysis

The following methods for metabolomic analysis were conducted by Human Metabolome Technologies (HMT), Inc. Frozen ground samples were transferred to a tube containing 600 μL methanol and 50 μM of an internal standard. After homogenization, 600 μL chloroform and 240 μL ultra-pure water were added to the homogenate, mixed well, and centrifuged at 2300 × *g* for 5 min at 4°C. The resultant water phases were ultra-filtrated using a Millipore Ultrafree-MC Centrifugal Filter Device 5 kDa (Millipore, Billerica, MA, United States) and then centrifuged at 9100 × *g* for 120 min at 4°C. The filtrates were dried, dissolved in 50 μL ultra-pure water, and subjected to capillary electrophoresis–time-of-flight mass spectrometry (CE-TOFMS) analysis using an Agilent CE-TOFMS system (Agilent, Palo Alto, CA, United States) following a previously described method (Soga and Neiger, 2000; Soga et al., 2002, 2003).

Cationic metabolites were analyzed as the following conditions: fused silica capillary (50 μm i.d. × 80 cm total length); run and rinse buffer, Cation Buffer Solution (p/n: H3301-1001); sample injection, pressure injection 50 mbar for 10 s; CE voltage, 27 kV; MS ionization, electrospray ionization (ESI) positive; MS capillary voltage, 4000 V; MS scan range, *m*/*z* 50–1000; sheath liquid, HMT Sheath Liquid (p/n: H3301-1020). Anionic metabolites were analyzed as the following conditions: fused silica capillary (50 μm i.d. × 80 cm total length); run and rinse buffer, Anion Buffer Solution (p/n: H3301-1021); sample injection, pressure injection 50 mbar for 25 s; CE voltage, 30 kV; MS ionization, ESI negative; MS capillary voltage, 3500 V; MS scan range, *m*/*z* 50–1000; sheath liquid, HMT Sheath Liquid (p/n: H3301-1020). Cation Buffer Solution, Anion Buffer Solution, and HMT Sheath Liquid were provided from HMT.

The peak detected by CE-TOFMS were extracted based on S/N >3.0 and analyzed to obtain the peak information including *m*/*z*, migration time, and peak area by MasterHands ver.2.17.1.11. Metabolites were identified by comparison of the migration time and *m*/*z* ratio with those of authentic standards, in which the difference of ±0.5 min and ±10 ppm was permitted, respectively. Relative peak areas were calculated from the internal standard, which was provided from HMT, and used the subsequent analysis for principal component analysis (PCA). Some of them were quantified by comparing their peak areas with those of the authentic standards. The metabolites detected in at least one sample were used for subsequent analyses. PCA was performed based on normalized data for the relative peak area to confirm reproducibility among replicates using the “prcomp” function in R software ver. 4.0.2.

### Statistical Analyses

Data were analyzed statistically using Welch’s *t*-test, Tukey’s honestly significant difference (HSD) test, or Dunnett’s test to determine the significant difference in the data among groups. *P-*values less than 0.05 were considered significant.

## Results

### Physiological Responses of Immature Tea Plants to Repeated Shading Treatments

**FIGURE 1:**
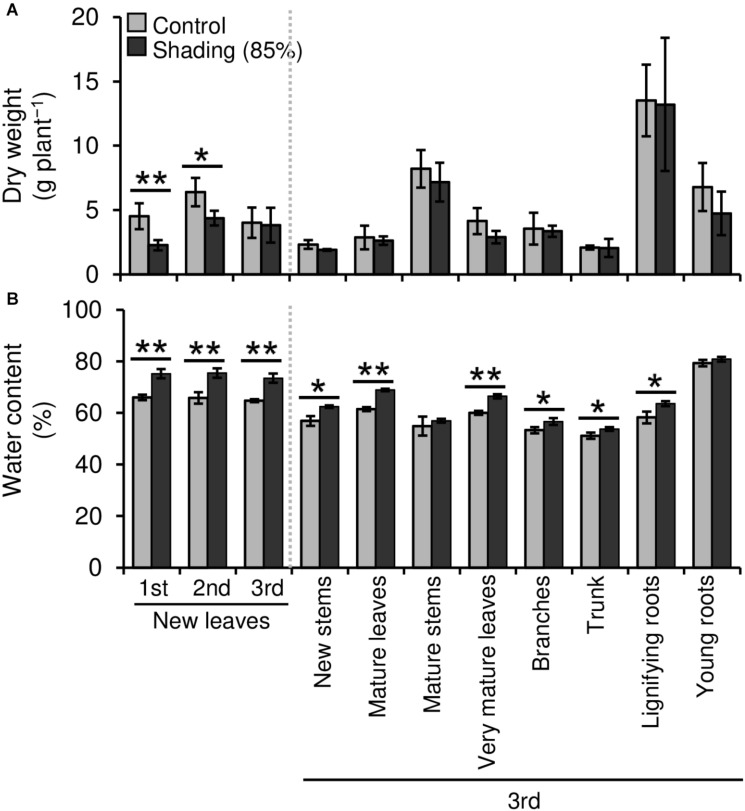
Effect of repeated shading on the dry weight **(A)** and water content **(B)** of whole organs of immature tea plants. The labels 1st, 2nd, and 3rd refer to the sequence of shading treatments. Gray and black bars indicate data for control and 85% shading treatments, respectively. Values are presented as means ± SD (1st: *n* = 6; 2nd: *n* = 5; 3rd: *n* = 3). Single and double asterisks indicate significant differences between the control and the 85% shading treatment at *P* < 0.05 and *P* < 0.01, respectively (Welch’s *t*-test).

**FIGURE 2:**
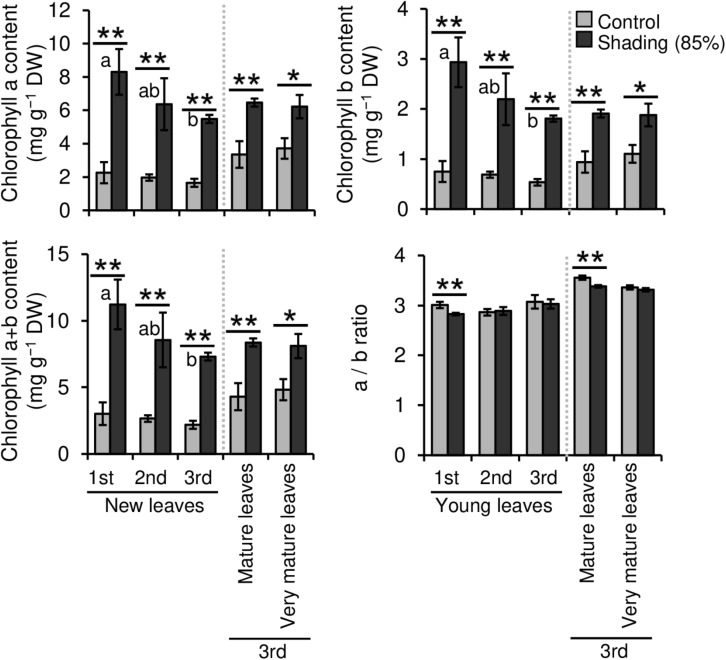
Effect of repeated shading on the chlorophyll content of leaves of immature tea plants. The labels 1st, 2nd, and 3rd refer to the sequence of shading treatments. Gray and black bars indicate data for control and 85% shading treatments, respectively. Values are presented as means ± SD (1st: *n* = 6; 2nd: *n* = 5; 3rd: *n* = 3). Single and double asterisks indicate significant differences between the control and the 85% shading treatment at *P* < 0.05 and *P* < 0.01, respectively (Welch’s *t*-test). Different letters indicate significant differences within each control or shading treatment (Tukey’s HSD test, *P* < 0.05).

**FIGURE 3:**
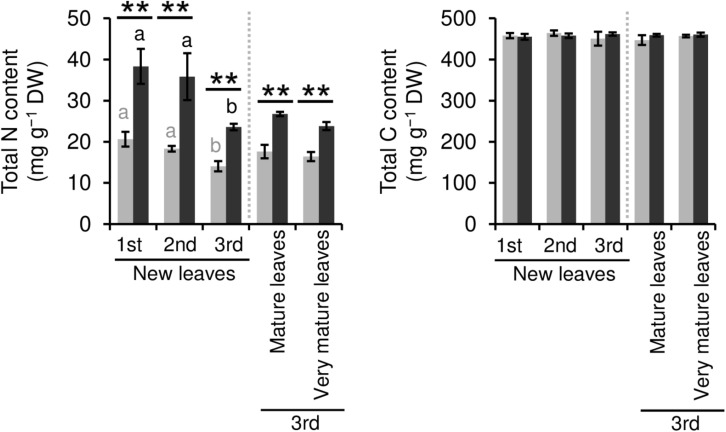
Effect of repeated shading on nitrogen (N) and carbon (C) contents of leaves of immature tea plants. The labels 1st, 2nd, and 3rd refer to the sequence of shading treatments. Gray and black bars indicate data for control and 85% shading treatments, respectively. Values are presented as means ± SD (1st: *n* = 6; 2nd: *n* = 5; 3rd: *n* = 3). Single and double asterisks indicate significant differences between the control and the 85% shading treatment at *P* < 0.05 and *P* < 0.01, respectively (Welch’s *t*-test). Different letters indicate significant differences within each control or shading treatment (Tukey’s HSD test, *P* < 0.05).

**FIGURE 4:**
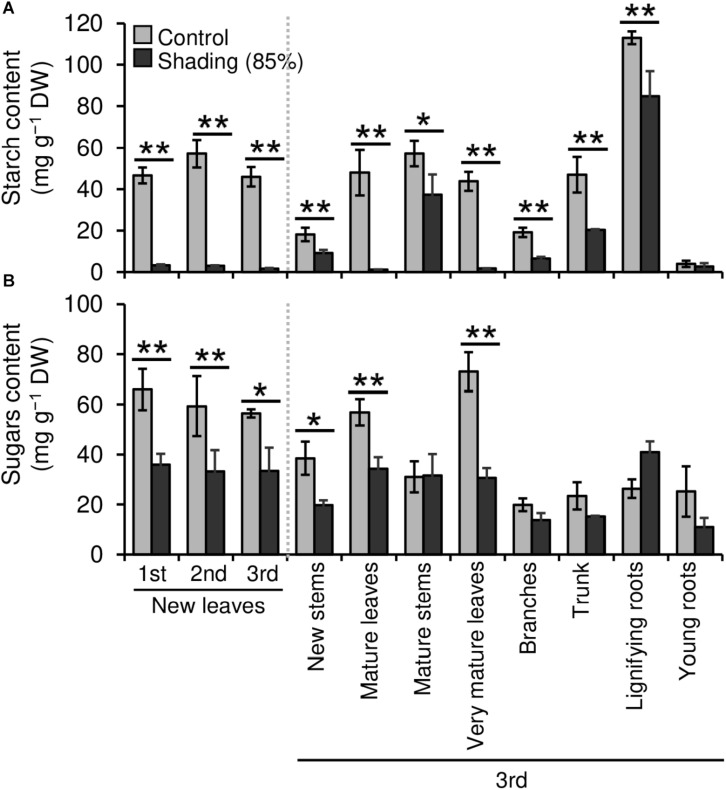
Effect of repeated shading on the non-structural carbohydrate content of whole organs of immature tea plants. Contents of starch **(A)** and sugar **(B)** as representative carbohydrates were determined in whole organs of immature tea plants. The labels 1st, 2nd, and 3rd refer to the sequence of shading treatments. Gray and black bars indicate data for control and the 85% shading treatments, respectively. Values are presented as means ± SD (1st: *n* = 6; 2nd: *n* = 5; 3rd: *n* = 3). Single and double asterisks indicate significant differences between the control and shading treatment at *P* < 0.05 and *P* < 0.01, respectively (Welch’s *t*-test). Different letters indicate significant differences between each control or shading treatment (Tukey’s HSD test, *P* < 0.05).

We evaluated the effect of repeated shading treatments on the physiological response of tea plants using immature pot-grown plants in a growth chamber. The growth of NL was significantly decreased after the first and second shading treatments, but no significant difference was observed after the third (Figure 1A). The water content of NL after each shading treatment and the majority of organs after the third shading treatment, except for MS and NR, was increased by shading (Figure 1B). The chlorophyll a and b contents of NL were increased by each shading treatment, and the degree of response was reduced by repeated shading (Figure 2). These responses in chlorophyll content were also observed in ML and VML after the third shading treatment (Figure 2). The chlorophyll a/b ratio of NL decreased only after the first shading treatment (Figure 2). Although the total N content of NL showed a similar response to that of chlorophyll content after shading, the total C content exhibited no significant differences in response to shading treatments (Figure 3). To assess the effect of shading on a detailed C source, we measured starch and sugar contents. The starch content of the majority of organs had decreased after the third shading treatment, but no significant differences in response to repeated shading were observed in NL (Figure 4A). Sugar content showed a similar response to shading as that of starch content; this was especially true for leaves, which are the main organs for photosynthetic C assimilation (Figure 4B).

**FIGURE 5:**
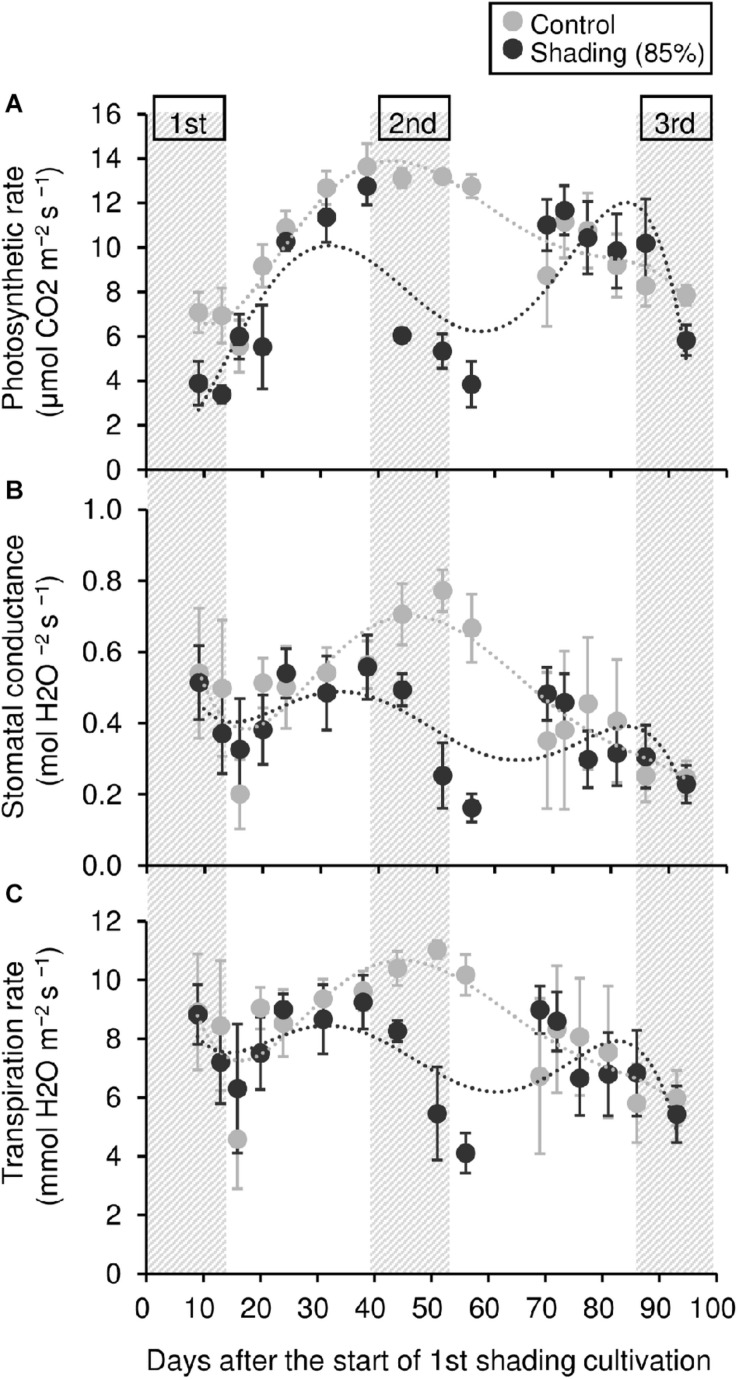
Effect of repeated shading on the photosynthetic ability of leaves of immature tea plants. Gray and black points indicate data for control and 85% shading treatments, respectively. The labels 1st, 2nd, and 3rd refer to the sequence of shading treatments. The gray area in the figure shows the shading treatment period. Values are presented as means ± SD (until the following number, 1st: *n* = 6; 2nd: *n* = 5; 3rd: *n* = 3). Photosynthetic rate **(A)**, stomatal conductance **(B)**, and transpiration rate **(C)** in the same leaves were measured with a Li-6400XT portable photosynthesis system.

**FIGURE 6:**
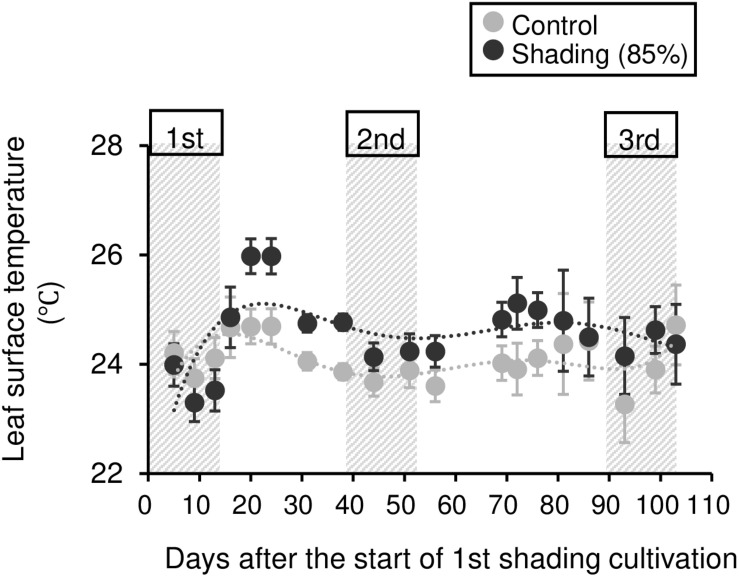
Effect of repeated shading on the leaf surface temperature of immature tea plants. Gray and black points indicate data for control and 85% shading treatments, respectively. The labels 1st, 2nd, and 3rd refer to the sequence of shading treatments. The gray area in the figure shows the shading treatment period. Values are presented as means ± SD (until the following number, 1st: *n* = 6; 2nd: *n* = 5; 3rd: *n* = 3). Leaf surface temperature in the same leaves was measured by infrared thermography.

To evaluate the physiological effect of shading treatments on immature tea plants, we investigated their photosynthetic ability. Photosynthetic rate tended to decrease during shading, especially during the second shading treatment (Figure 5A). Similar responses were observed for stomatal conductance and transpiration rate (Figures 5B,C). In shaded plants, the leaf surface temperature was increased during the first shading treatment compared with that of control plants (Figure 6).

### Effect of Repeated Shading Treatments on the Starch Content of Mature Tea Plants

**FIGURE 7:**
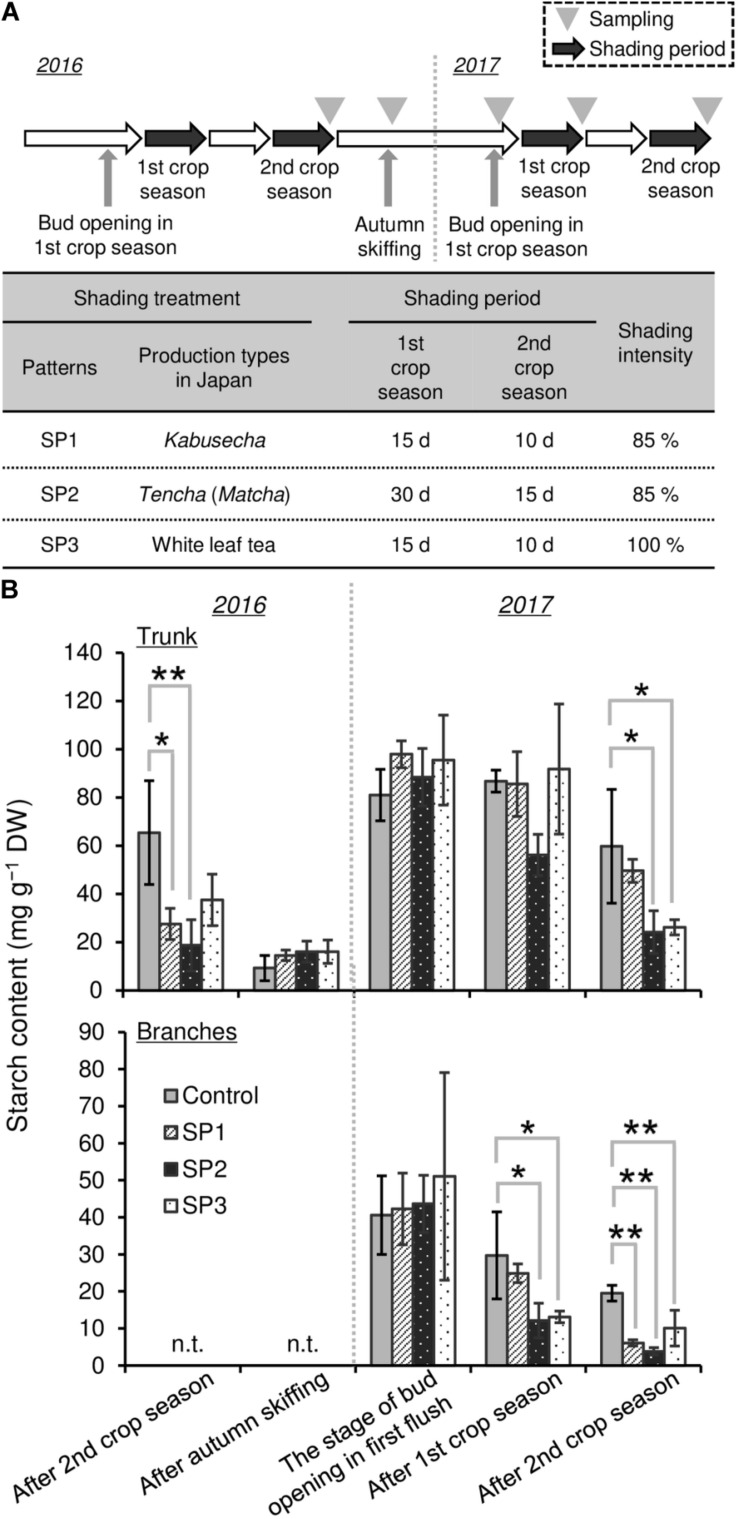
Effect of three repeated shading treatments on seasonal starch contents of sink organs of mature tea plants in the field. **(A)** The experimental design. **(B)** Starch contents of trunks and branches. Values are presented as means ± SD (*n* = 3). Single and double asterisks indicate significant differences between the control and each shading treatment at *P* < 0.05 and *P* < 0.01, respectively (Dunnett’s test).

To evaluate the effect of shading on a C resource in mature tea plants in the field, we conducted three types of shading treatments (SP1, SP2, and SP3, shown in Figure 7A) and investigated the seasonal response of starch content of BR and TR, the main sink organs of tea plants. A decrease in starch content owing to shading treatments, especially SP2, was observed in trunks after treatment in the second crop season but not in the first crop season (Figure 7B). Starch content recovered in autumn and remained at original levels until the following first crop season. In branches, a decrease in starch content due to shading was observed in SP2 and SP3 after the first crop season and in all shading treatments after the second crop season (Figure 7B).

### Effect of Long-Term Repeated Shading on the Yield and Photosynthetic Ability of Mature Tea Plants in Mid-Summer

**FIGURE 8:**
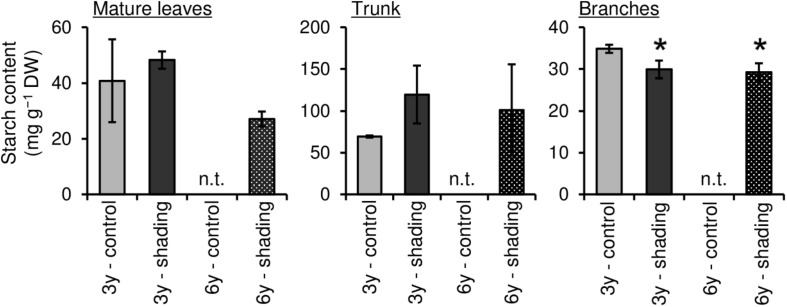
Effect of repeated shading on starch contents of each organ of mature tea plants at the bud-opening stage in the first crop season. “3y–shading,” shading was applied only in the first crop season for 3 consecutive years until the examined year; “6y–shading,” shading was applied in the first- and second-crop seasons for 6 consecutive years until the examined year. Uncovered tea ridges, ridges adjacent to the shade-treated tea ridges and used as controls (“3y–control” and “6y–control”, respectively). We could not sample 6y–control plants at the time of bud opening; for this stage, we instead used the 3y–control in its place for comparisons with 6y–shading. Values are presented as means ± SD (*n* = 3). Single asterisks indicate significant differences between the 3y-control and each shading treatment at *P* < 0.05 (Dunnett’s test). n.t., not tested.

**FIGURE 9:**
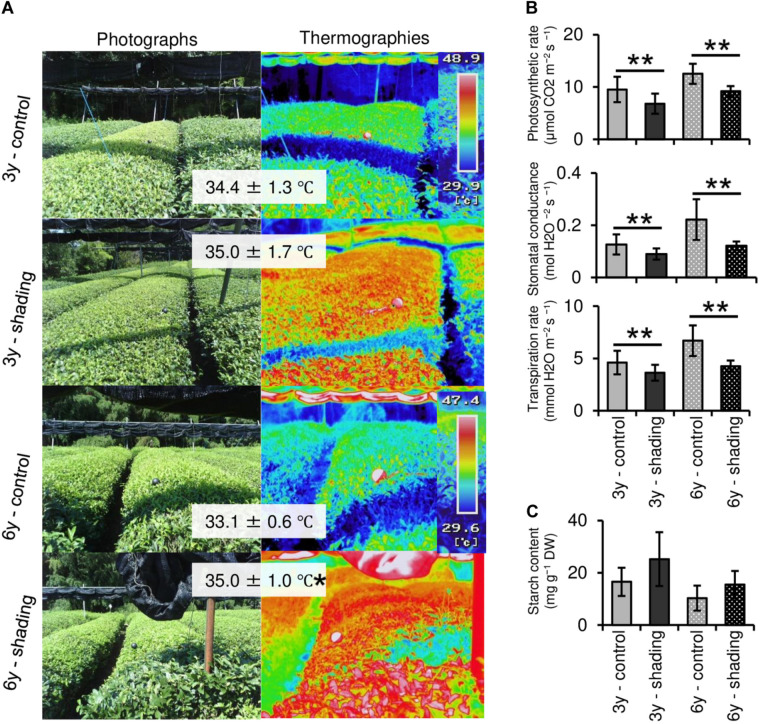
Effect of repeated shading on the canopy temperature and photosynthetic ability of mature leaves of mature tea plants in the mid-summer season. **(A)** Canopy temperature of tea ridges. The left and right images are photographs and thermographs, respectively. The values in gray boxes are the mean ± SD (*n* = 3–13) canopy temperature. **(B)** Photosynthetic rate, stomatal conductance, and transpiration rate in the same mature leaves measured with a Li-6400XT portable photosynthesis system. **(C)** Starch content of mature leaves. Values in **(B,C)** are presented as means ± SD (*n* = 5 and *n* = 4, respectively). “3y–shading,” shading was applied only in the first crop season for 3 consecutive years until the examined year; “6y–shading,” shading was applied in the first- and second-crop seasons for 6 consecutive years until the examined year. Uncovered tea ridges, ridges adjacent to the shade-treated tea ridges and used as controls (“3y–control” and “6y–control,” respectively). Single and double asterisks indicate significant differences among shading treatments at *P* < 0.05 and *P* < 0.01, respectively (Welch’s *t*-test).

To evaluate the effect of long-term repeated shading, we used tea ridges subjected to excessive stress induced by repeated shade cultivation in a previous study by Takemoto and Hayashi (2019). In these tea ridges, yield components, especially the number of shoots in the first crop season, were decreased by long-term shade cultivation for 6 consecutive years (Table 1). Although the starch content of branches decreased in response to repeated shading at the bud opening stage of the first crop season, the starch content of ML and TR was unchanged (Figure 8). The canopy temperature increased in response to shading treatments only for 6y-shading (Figure 9A). Photosynthetic rate, stomatal conductance, and transpiration rate were decreased by 3y-shading and 6y-shading compared with those of each control (Figure 9B), but the relative changes were larger for 6y-shading. However, the starch content of ML did not significantly change (Figure 9C).

**TABLE 1:** Effect of repeated shading on yield components in the first crop season in mature tea plants in the field.

Repeated shading years	Treatment	Shoot length (cm)	Number of new leaves	Number of shoots (stem 400 cm^–2^)	Weight of 100 shoots (g FW)	Ratio of dormant shoots (%)	Theoretical yield (kg 10 a^–1^)
3 years	Control	5.3 ± 0.3	3.4 ± 0.1	45.3 ± 11.2	100.3 ± 8.8	93.5 ± 1.0	1127.9 ± 233.0
	Shading	4.1 ± 0.7	3.2 ± 0.1	49.3 ± 8.5	76.6 ± 10.1*	85.8 ± 5.7	930.7 ± 49.1
6 years	Control	6.3 ± 0.9	3.5 ± 0.1	62.7 ± 4.0	97.3 ± 15.1	89.1 ± 6.1	1526.7 ± 265.5
	Shading	4.2 ± 1.2	3.0 ± 0.2	31.7 ± 8.5*	84.9 ± 11.6	92.9 ± 6.4	684.4 ± 267.6*

*Values are presented as means ± SD (n = 3). Asterisks indicate significant differences among shading treatments at P < 0.05 (Welch’s t-test).*

### Metabolomic Detection of Candidate Biomarkers Reflecting the Status of Overstressed, Shaded Tea Plants

A metabolomic analysis was conducted to detect candidate biomarkers reflecting the status of plants in response to repeated shading. First, to capture basic changes in the metabolome, we quantified 74 metabolites using whole organs after the second shading treatment collected from immature tea plants grown in a growth chamber. We focused on the variation in amino acids, which are important metabolites of tea. Broad variation in amino acid contents, especially in major sink organs, such as BR and TR, was observed in response to shading (Supplementary Figure 2). In TR, the contents of arginine and asparagine, which are major N assimilation metabolites, were markedly changed (Supplementary Table 1). In addition, we used TR as the focus for metabolomic analysis of shade-treated plants in the field because TR are easily harvested, and the risk of contamination is low.

**FIGURE 10:**
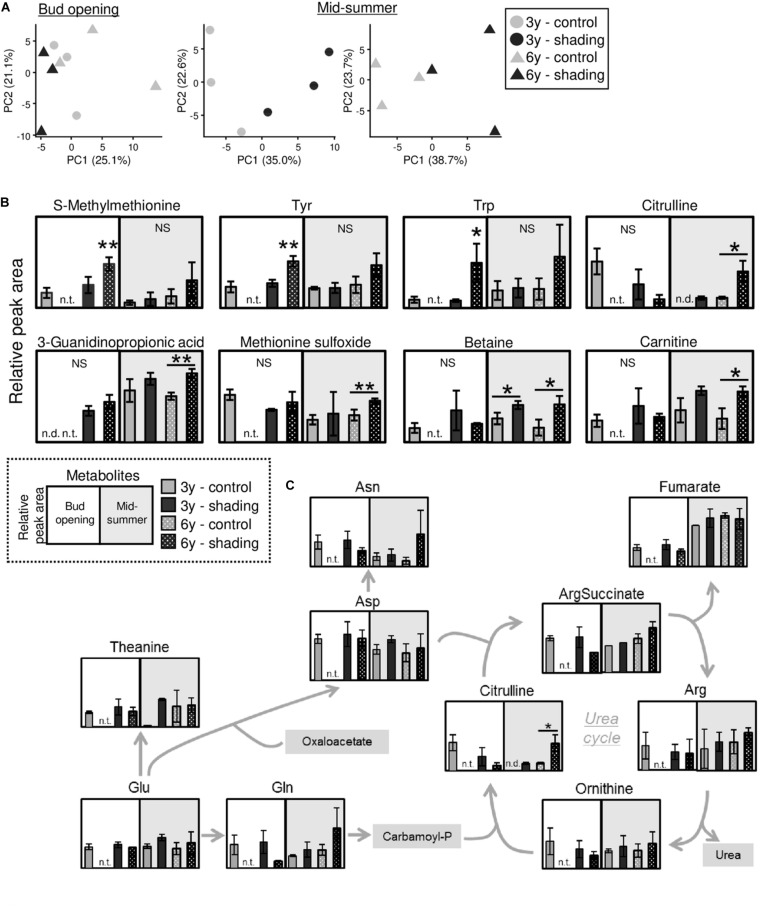
Metabolomic analysis of trunks of mature tea plants subjected to repeated shading at the bud-opening stage in the first-crop and mid-summer seasons. **(A)** Biplots from a principal component analysis of detected metabolites. **(B)** Metabolites significantly altered by repeated shading cultivation at the bud-opening stage in the first-crop or mid-summer season. **(C)** Pathway view of metabolites involved in nitrogen assimilation and the urea cycle. White and gray points represent data for bud opening and the mid-summer season, respectively. At the bud-opening stage, single and double asterisks indicate significant differences between the 3y-control and each shading treatment at *P* < 0.05 (Dunnett’s test). In the mid-summer season, single and double asterisks indicate significant differences among shading treatments at *P* < 0.05 and *P* < 0.01, respectively (Welch’s *t*-test). n.t., not tested; n.d., not detected.

Using tea plants that were overstressed because of repeated shade cultivation, we conducted a metabolomic analysis of TR at the bud-opening stage in the first crop season and mid-summer. In total, 133 metabolites (76 in the cation mode and 57 in the anion mode) and 146 metabolites (91 in the cation mode and 55 in the anion mode) were detected by metabolomic analysis at the bud-opening stage of the first-crop and mid-summer seasons, respectively (Supplementary Tables 2, 3). A PCA was performed to assess the reproducibility of the metabolome in this study. Under the same conditions, data for all three triplicates corresponded closely, and shaded and unshaded (control) plants were separated by the first principal component (Figure 10A). Statistical analysis identified candidate biomarkers that were changed in plants affected by shading. S-methylmethionine, tyrosine, and tryptophan were more highly accumulated in the 6y-shading plants at the bud-opening stage of the first crop season (Figure 10B). In addition, citrulline, 3-guanidinopropionic acid, methionine sulfoxide, betaine (glycine betaine), and carnitine were highly accumulated in the 6y-shading plants in the mid-summer season. In the N assimilation pathway and urea cycle, the majority of metabolites, except citrulline, were unchanged in response to repeated shading (Figure 10C).

## Discussion

Shade cultivation, a traditional Japanese tea cultivation system, is used to produce high-quality Japanese green tea products, such as *Matcha* and *Gyokuro*. Shading markedly changes the phenotypes of tea plants, especially leaf color, which becomes dark green because of chloroplast enlargement, increased chlorophyll content, and up-regulation of genes involved in the chlorophyll biosynthesis pathway in response to limited sunlight (Sano et al., 2018; Liu et al., 2020). Limiting irradiation substantially stresses plant growth, and repeated shading of tea plants potentially reduces their health. Therefore, we conducted a pot experiment as a basic model to evaluate physiological responses of tea plants to repeated shading treatments. In the present study, an increase in chlorophyll a and b contents of NL was observed in response to shading, but these effects were reduced by repeated shading (Figure 2). N content, which directly affects leaf color, was reduced by repeated shading as well (Figure 3). These results suggest that the response of tea plants to shading is dependent on N nutrient status. In contrast, repeated shading had no observable effects on the contents of other mineral elements, such as Mg and Fe, that contribute to the green color of leaves (Supplementary Figure 4). Thus, these results highlight the importance of regulating the application of N in shaded-tea cultivation.

The NSC content of the majority of organs was decreased in response to shading. A decrease in leaves was observed, but no significant decline was observed upon repeated shading (Figure 4). Tree-specific sink organs not associated with photosynthesis, such as BR, TR, and LR, are strongly affected by reduced NSC content. Repeated shade cultivation causes the marked decrease in NSC content to continue and may have a major impact on the productivity and quality of tea plants. NSCs are directly or indirectly involved as respiratory substrates in all plant primary and secondary metabolic processes (Hartmann and Trumbore, 2016). Indeed, photosynthetic rate, stomatal conductance, and transpiration rate tended to decrease during shading (Figure 5), and leaf surface temperature increased during cultivation compared with that of the control after shading (Figure 6). The observed physiological responses, such as decreased photosynthesis and reduced NSC content, in shaded tea plants, were indicative of the accumulation of damage to the plant.

To evaluate the effect of shading on a C resource in mature tea plants in the field, we investigated the physiological response of the NSC content of major sink organs under three shading treatments. A marked decrease in starch content was observed in BR and TR of mature tea plants after the second crop season, with shading intensity and duration having strong impacts, but these effects disappeared the following year (Figure 7). We also measured the starch content of tea plants subjected to excessive stress due to repeated shade cultivation that were studied previously by Takemoto and Hayashi (2019). At the bud-opening stage in the first crop season, the starch content of branches decreased in response to repeated shading but was unchanged in ML and TR (Figure 8). According to Li et al. (2020), who performed metabolic profiling, C metabolism in leaves is inhibited by long-term shading. Therefore, carbon metabolism in overstressed tea plants may be inhibited throughout the entire plant, not just the branches, even if no effect on final starch content is observed. In addition, Suzuki et al. (2013b) have reported that a lack of starch in the major sink organs of mature tea plants during the bud-opening stage leads to a decrease in the yield and quality of the next first-crop season. These results suggest that the reduction in NSC content caused by shading at the bud-opening stage might be reversed in mature tea plants by returning to conventional cultivation practice, whereas overstressed plants, which are damaged by repeated shading, are unable to recover after a return to conventional cultivation until the next crop season.

Photosynthetic rate, stomatal conductance, and transpiration rate tended to decrease during shading treatments (Figure 5), while leaf surface temperature increased during cultivation after shading in immature tea plants (Figure 6). This phenomenon of an increased leaf surface temperature suggests that “the release of the shading condition” causes some injury, namely, high light stress to low light-adapted leaves of shaded individuals, resulting in the observed phenotype of an increased leaf surface temperature because shaded tea plants undergo sudden exposure to high light at the end of the treatment due to shade removal. The initial exposure of low light-adapted plants to high light conditions, may result in the enhanced generation of reactive oxygen species (ROS), and subsequent induction of ROS-scavenging and detoxification systems. To acclimate high light conditions, low light-adapted plants modulates the photosynthetic light reactions, including photoinhibition (Athanasiou et al., 2010), activation of cyclic electron flow (Yamori and Shikanai, 2016), re-routing of excess electrons to alternative electron sinks (Apel and Hirt, 2004), and the induction of non-photochemical quenching (NPQ) for the dissipation of excess absorbed energy (Ruban, 2016). Actually, Sano et al. (2020) have reported that shaded tea plants suffer from oxidative damage caused by high light stress. Takemoto and Hayashi (2019) observed a higher temperature on the canopy surface in mid-summer in response to repeated shading in overstressed tea plants. Similarly, we measured the mid-summer canopy temperature of the overstressed tea ridges in which shade cultivation had been withheld in the surveyed year. The increase in canopy surface temperature that resulted from shading treatments was observed only in 6y-shading plants (Figure 9A). Based on the cooling effect of the transpiration process, canopy temperature has been proposed as an indicator of plant water stress since the 1960s (Tanner, 1963). In addition, canopy temperature can be used to estimate varietal differences in stomatal conductance in rice (Takai et al., 2010; Fukuda et al., 2018). Thus, transpiration decreases if plant water stress increases, and plant temperature may exceed the air temperature (Wang et al., 2010). In the overstressed tea plants in the present study, photosynthetic rate, stomatal conductance, and transpiration rate were decreased by repeated shading treatments (Figure 9B). These results suggest that the increase in canopy temperature observed in overstressed mature tea plants was also caused by a decrease in transpiration ability.

To detect candidate biomarkers reflecting the effect of repeated shade cultivation, we conducted a metabolomic analysis on the trunks of overstressed tea plants at the bud-opening stage in the first crop and mid-summer seasons. Several metabolites were significantly changed in individuals affected by shade cultivation (Figure 10B). In the mid-summer season, in particular, citrulline and betaine (glycine betaine) were remarkably highly accumulated in response to long-term shade cultivation in 6y-shading plants. Citrulline, which is a novel compatible solute, is highly accumulated in response to drought in watermelon and contributes to oxidative stress tolerance under drought as a hydroxyl radical scavenger (Akashi et al., 2001). Enhanced glycine betaine accumulation in transgenic maize improves drought tolerance (Quan et al., 2004). In addition, glycine betaine might alleviate the effect of high-temperature stress, as the extent of heat-shock proteins has been found to be significantly reduced in transgenic Arabidopsis accumulating glycine betaine (Hayashi et al., 1998; Sakamoto and Murata, 2002). The accumulation of these compatible solutes involved in water stress in the mid-summer season may be an indicator of the overstressed status of mature tea plants subjected to repeated shade cultivation. Analysis of the profiles of the candidate biomarkers might enable prediction of whether shade cultivation is feasible in the next crop season.

In the present study, we demonstrated that shade cultivation caused a decrease in NSC content and an increase in leaf surface temperature as a result of a decline in photosynthetic ability. An increase of several degrees in canopy temperature in the mid-summer season, which required a high transpiration rate, was observed in overstressed mature tea plants subjected to repeated shade cultivation. Metabolomic analysis identified several candidate biomarkers, such as citrulline and glycine betaine, that were significantly changed in response to shade cultivation. These physiological changes may be useful indicators of the status of overstressed tea plants grown under repeated shade cultivation.

## Data Availability Statement

The original contributions presented in the study are included in the article/Supplementary Material, further inquiries can be directed to the corresponding author.

## Author Contributions

HY, YT, KU, SM, YO, and TI performed the pot experiment in the growth chamber. TS and TT cultivated and managed the mature tea plants for shading tests in the field. KU, YO, YT, and TT measured leaf surface and canopy temperatures by thermography and determined photosynthetic ability. YT, KU, SM, YO, and TS performed the starch and sugar analyses. HY performed the mineral analysis. HY, YT, and TI analyzed the metabolomic data. HY and TI performed most of the data visualization and writing. AM supervised the research and edited the manuscript. TS, TT, AM, and TI designed the research and acquired the funding. All authors approved the manuscript.

## Conflict of Interest

The authors declare that the research was conducted in the absence of any commercial or financial relationships that could be construed as a potential conflict of interest.

## Supplementary Material

Supplementary Figure 1 Overview of repeated shading treatments of pot-grown immature tea plants in a growth chamber. **(A)** Experimental design of repeated shading treatments. **(B)** Separated organs of sampled immature tea plants.

Supplementary Figure 2 Effect of repeated shading on metabolomic profiles of major organs of pot-grown immature tea plants in a growth chamber. Heatmap visualization of normalized quantitative metabolite data is shown for the following organs: NL, new leaves; ML, mature leaves; BR, branches; TR, trunks; NR, new roots; LR, lignified roots.

Supplementary Figure 3 Experimental design to evaluate the effect of repeated shading on tea ridges exhibiting accumulated damage due to shading.

Supplementary Figure 4 Effect of repeated shading on iron (Fe) and magnesium (Mg) contents of new leaves of immature tea plants.

Supplementary Table 1 Metabolites quantified by CE-TOFMS in major organs of pot-grown immature tea plants subjected to repeated shading in a growth chamber.

Supplementary Table 2 Metabolites detected by CE-TOFMS in trunks of mature tea plants under continuous shading cultivation at the bud-opening stage in the first crop season.

Supplementary Table 3 Metabolites detected by CE-TOFMS in trunks of mature tea plants under continuous shading cultivation in the mid-summer season.
