# Supplementary material for: Phenotypic Markers Reflecting the Status of Overstressed Tea Plants Subjected to Repeated Shade Cultivation
Source: Front Plant Sci. 2020 Nov 6;11:556476. doi: 10.3389/fpls.2020.556476 (PMC7677308; doi:10.3389/fpls.2020.556476)
Supplement: Supplementary Figure 1 — Overview of repeated shading treatments of pot-grown immature tea plants in a growth chamber. (A) Experimental design of repeated shading treatments. (B) Separated organs of sampled immature tea plants. [file Presentation_1.PPTX]

## Slide 1
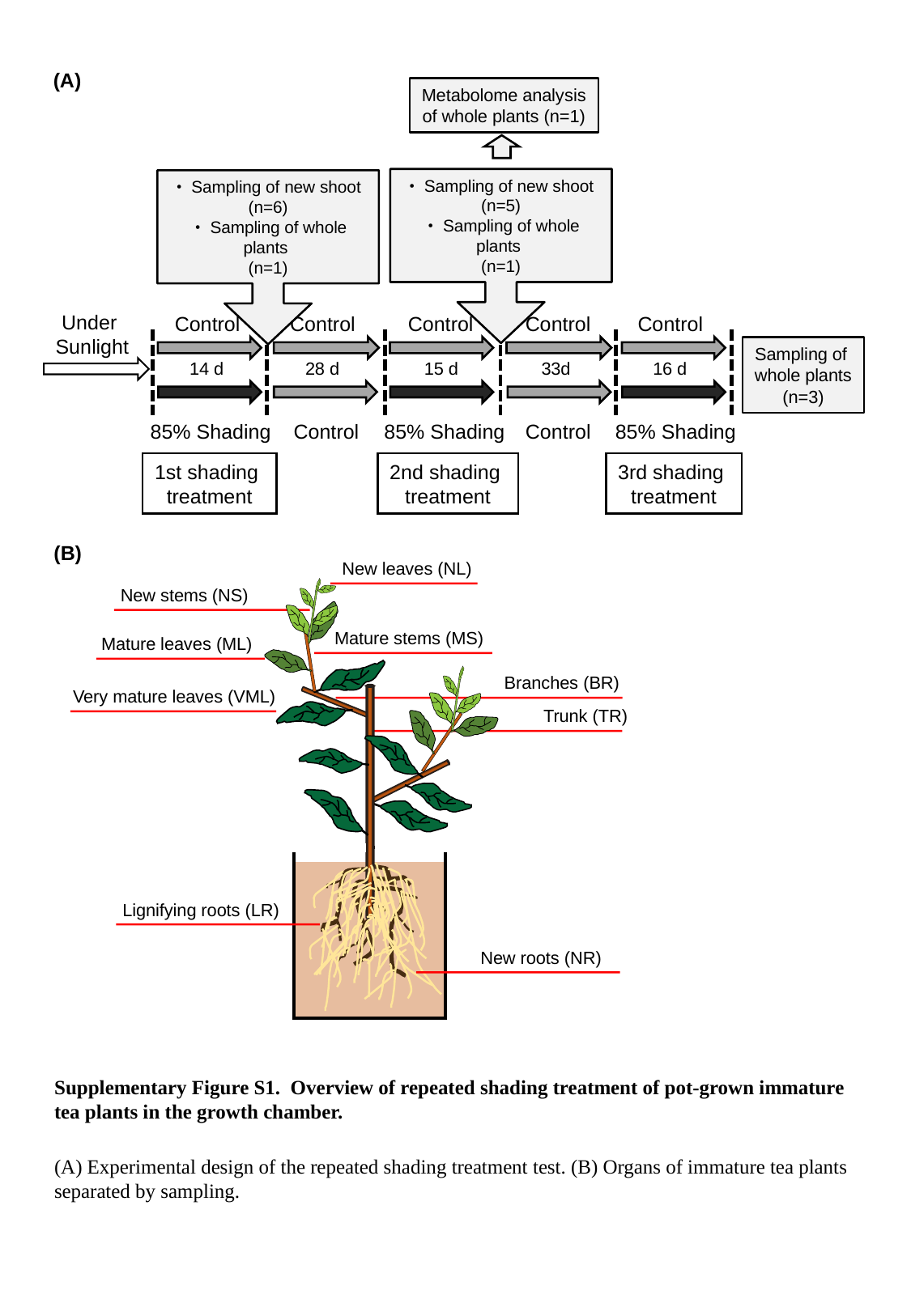

(A)
Metabolome analysis
of whole plants (n=1)
・Sampling of new shoot
(n=5)
・Sampling of whole plants
(n=1)
・Sampling of new shoot
(n=6)
・Sampling of whole plants
(n=1)
Under
Sunlight
Control
Control
Control
Control
Control
Sampling of
whole plants
(n=3)
14 d
28 d
15 d
33d
16 d
85% Shading
Control
85% Shading
Control
85% Shading
1st shading
treatment
2nd shading
treatment
3rd shading
treatment
(B)
New leaves (NL)
New stems (NS)
Mature stems (MS)
Mature leaves (ML)
Branches (BR)
Very mature leaves (VML)
Trunk (TR)
Lignifying roots (LR)
New roots (NR)
Supplementary Figure S1. Overview of repeated shading treatment of pot-grown immature tea plants in the growth chamber.
(A) Experimental design of the repeated shading treatment test. (B) Organs of immature tea plants separated by sampling.

## Slide 2
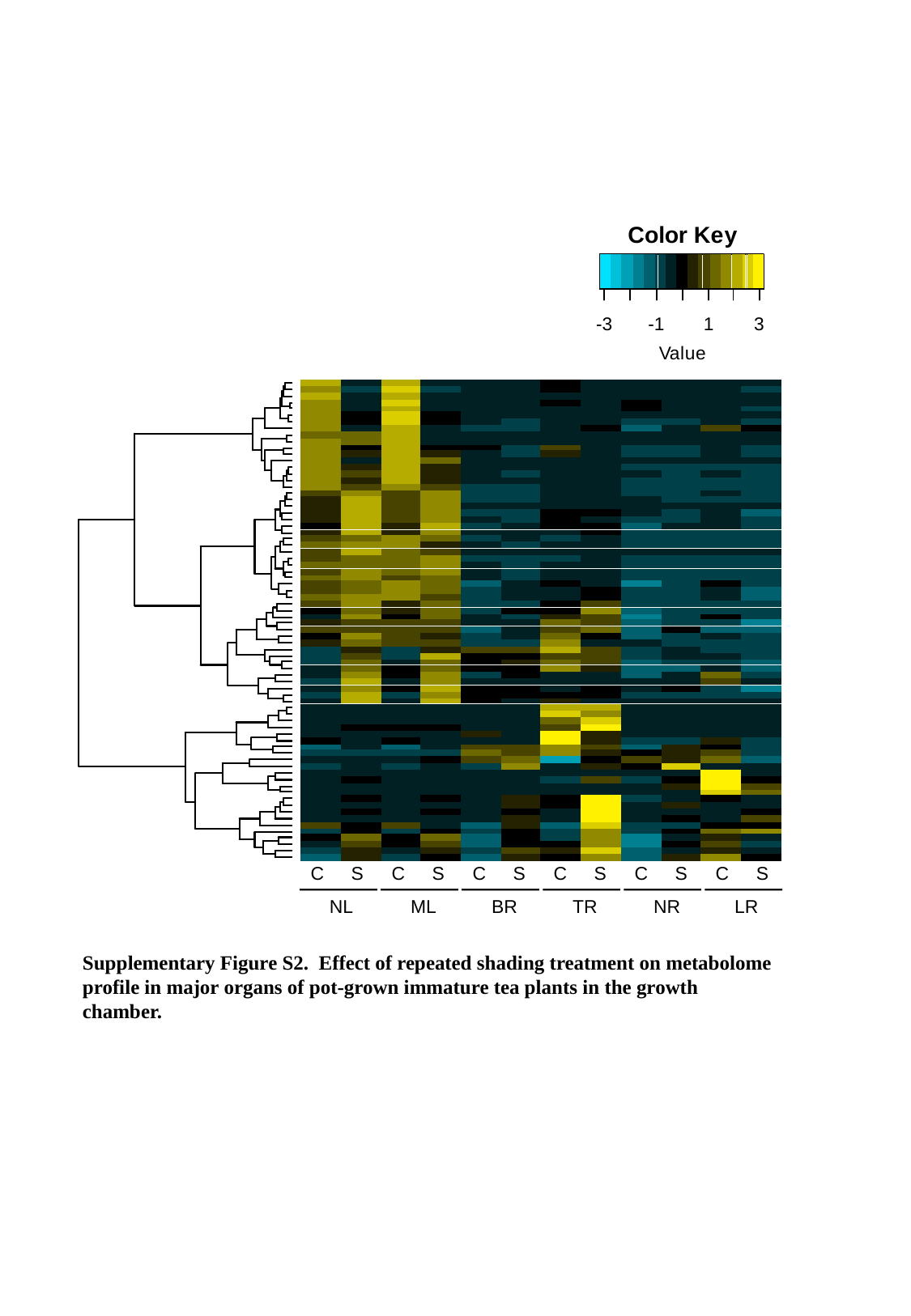

C
S
NL
C
S
ML
C
S
BR
C
S
TR
C
S
NR
C
S
LR
Supplementary Figure S2. Effect of repeated shading treatment on metabolome profile in major organs of pot-grown immature tea plants in the growth chamber.

## Slide 3
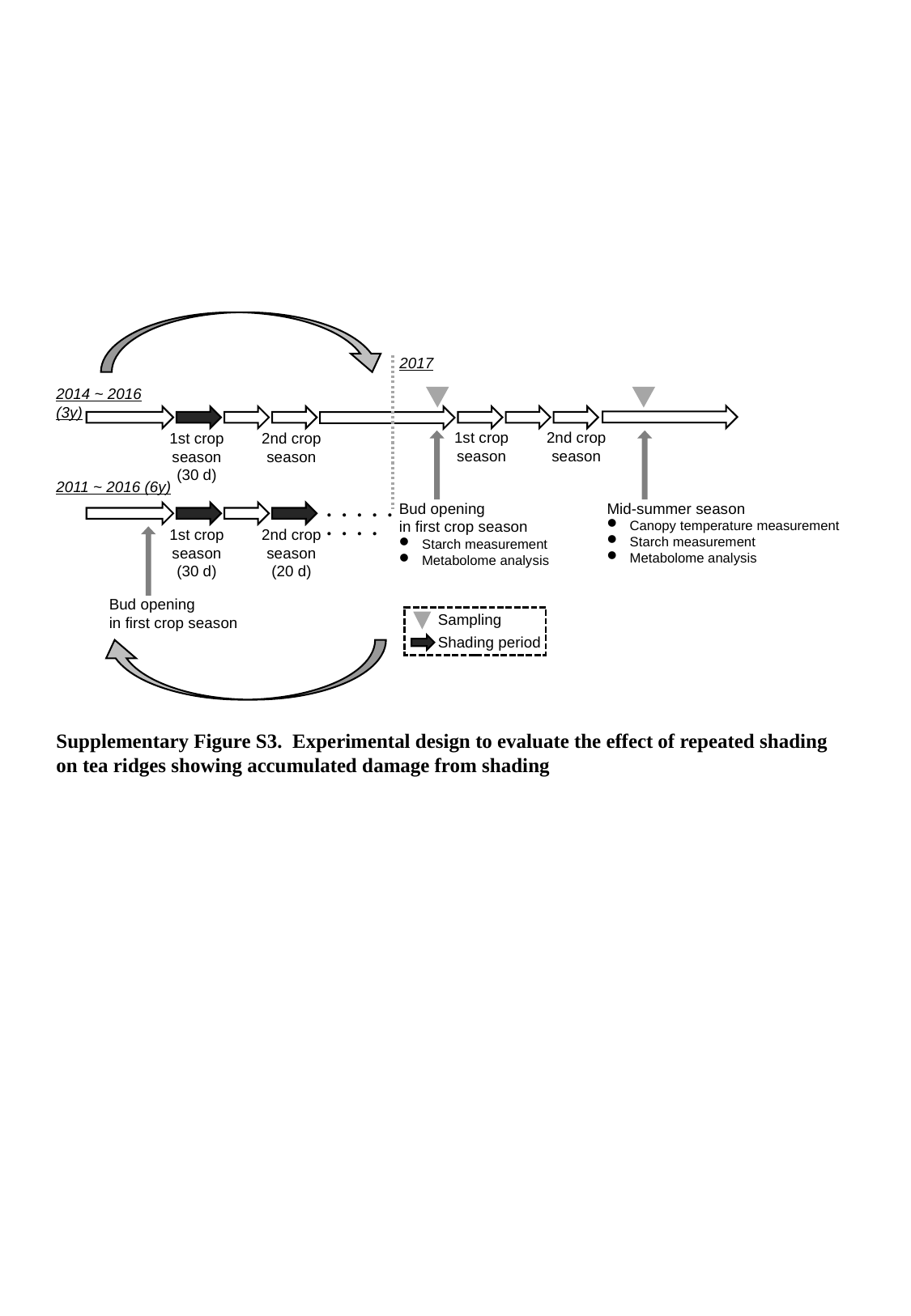

2017
2014 ~ 2016 (3y)
1st crop season
2nd crop season
1st crop season
(30 d)
2nd crop season
2011 ~ 2016 (6y)
Bud opening
in first crop season
Starch measurement
Metabolome analysis
Mid-summer season
Canopy temperature measurement
Starch measurement
Metabolome analysis
・・・・・・・・・
1st crop season
(30 d)
2nd crop season
(20 d)
Bud opening
in first crop season
Sampling
Shading period
Supplementary Figure S3. Experimental design to evaluate the effect of repeated shading on tea ridges showing accumulated damage from shading

## Slide 4
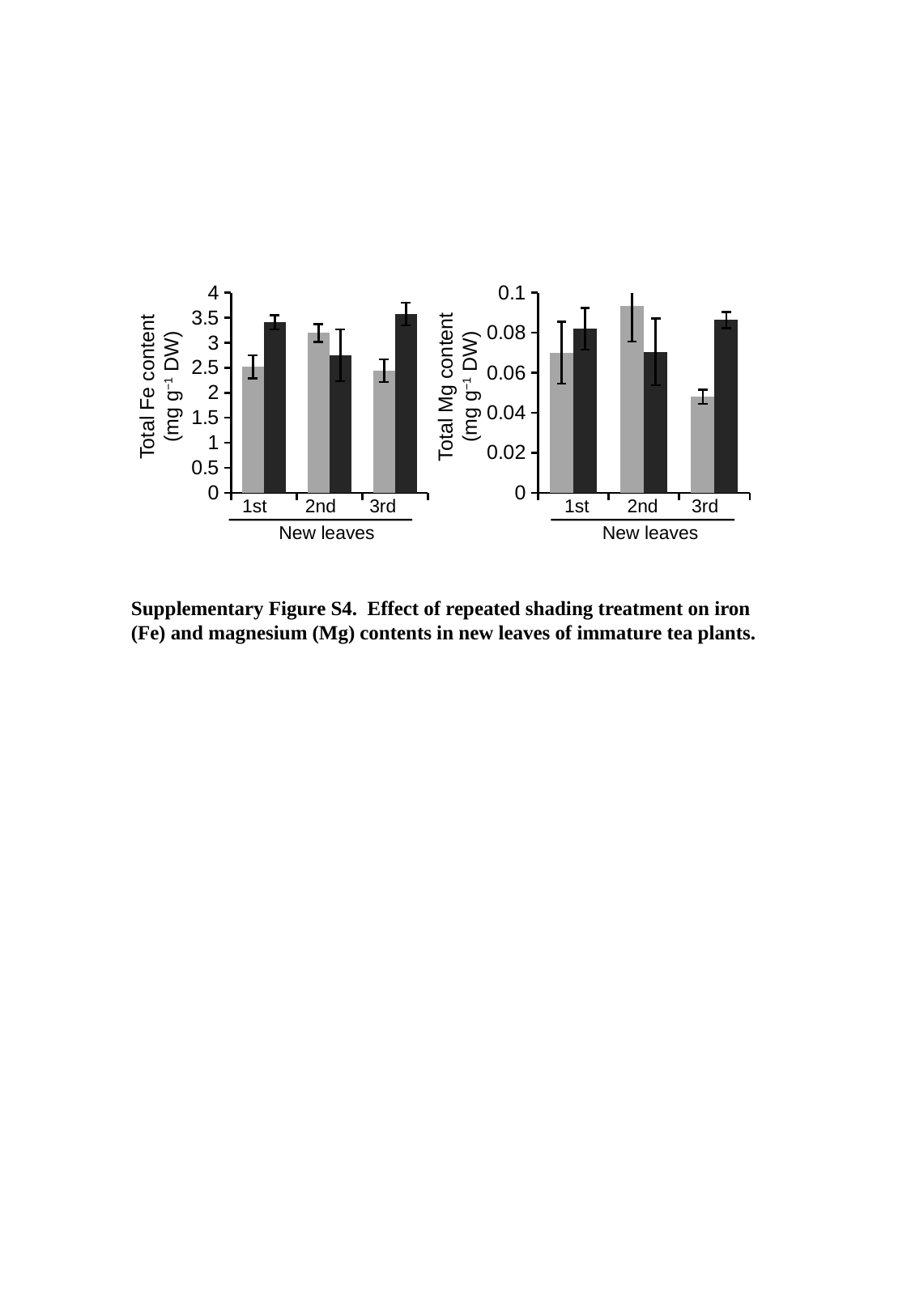

### Chart
| Category | Control | 85% Shading |
|---|---|---|
### Chart
| Category | Control | 85% Shading |
|---|---|---|Total Fe content
(mg g−1 DW)
Total Mg content
(mg g−1 DW)
1st
2nd
3rd
1st
2nd
3rd
New leaves
New leaves
Supplementary Figure S4. Effect of repeated shading treatment on iron (Fe) and magnesium (Mg) contents in new leaves of immature tea plants.
